# Lifestyle determinants of diabetes mellitus amongst people living with HIV in the Eastern Cape province, South Africa

**DOI:** 10.4102/phcfm.v14i1.3256

**Published:** 2022-05-12

**Authors:** Nokwanda E. Bam, Wezile Chitha, Jafta Ntsaba, Sibusiso C. Nomatshila, Teke Apalata, Sikhumbuzo A. Mabunda

**Affiliations:** 1 Department of Nursing, Faculty of Health Sciences, North-West University, Mahikeng, South Africa; 2 Health Systems Innovation Unit, Faculty of Health Sciences, University of the Witwatersrand, Johannesburg, South Africa; 3 Department of Nursing, Faculty of Health Sciences, Walter Sisulu University, Mthatha, South Africa; 4 Department of Public Health, Faculty of Health Sciences, Walter Sisulu University, Mthatha, South Africa; 5 Department of Laboratory Medicine and Pathology, Faculty of Health Sciences, Walter Sisulu University, Mthatha, South Africa; 6 The George Institute for Global Health, Faculty of Health Sciences, University of New South Wales, Sydney, Australia

**Keywords:** lifestyle, people living with HIV, PLWHIV, type 2 DM, HIV/AIDS, patients, ARV

## Abstract

**Background:**

Type 2 diabetes mellitus (DM) has serious consequences for those affected. Little is documented on the lifestyle determinants of type 2 DM in people living with human immunodeficiency virus (PLWHIV).

**Aim:**

This study aimed to assess the lifestyle determinants of type 2 DM amongst PLWHIV who were on antiretroviral treatment (ARV).

**Setting:**

This study was undertaken in 10 community health clinics and 140 clinics in South Africa’s Eastern Cape province.

**Methods:**

This case control study was undertaken amongst PLWHIV who were on ARV in OR Tambo district.

**Results:**

Cases and controls showed statistically significant differences on the duration of time on ARV (*p* < 0.0001), vigorous work (*p* = 0.019), participation in moderate sport (*p* = 0.007) and consuming daily fruit and vegetable servings (*p* = 0.021). Those reporting to be on ARVs for 6 to 10 years were three times more likely to be diabetic than those who had only been on ARV for a year or less (odds ratio [OR] = 3.0; *p* = 0.017) and in comparison, to participants who reported having one serving, participants who had four fruit and vegetable servings daily were 3.2 times more likely to be diabetic (OR = 3.2; *p* = 0.002).

**Conclusion:**

This study revealed significant nutritional imbalances on fruit and vegetable servings and on participation in moderate sport resulting in poor diabetic control. Routine screening and measurements need to focus on dietary and physical lifestyle determinants of type 2 DM in order to counsel patients on ARV on balanced nutrition and optimise outcomes in the quality care of PLWHIV.

## Background

Age, female gender, diet, smoking and obesity are independently and collectively associated with higher risk of type 2 diabetes mellitus (DM).^1^ This is besides the established association between combined antiretroviral treatment (ARV) and DM.^2,3,4,5^ Indicators of obesity such as weight, body fat percentage, waist–hip circumference and body mass index (BMI) that are above the designated values are known to forecast DM.^4,6^

Human immunodeficiency virus (HIV) is hypothesised to predispose patients to DM through several mechanisms, some of which are not as yet completely understood.^7^ A 2014 United States (US) study^8^ found the prevalence of DM to be higher in overweight and HIV infected patients (3.9% and 12.7%) compared with amongst non-HIV infected patients (3.1% and 7.8%).^8^ Furthermore, type 2 DM has been reported to be more prevalent in people living with HIV (PLWHIV) at about 5.0% higher amongst females; 4.1% higher in the age-group between 20 and 44 years and 3.5% higher amongst non-obese PLWHIV when compared with the general population.^4,9,10^

It is further hypothesised that viral load fluctuations cause life-long inflammation, which in turn leads to DM because of the resultant insulin resistance; patients with low levels of cluster of differentiation 4 (CD4) count were reported to have higher problems with glucose metabolism than those with higher CD4 counts and through poorly understood mechanisms ARVs are highly associated with the prevalence of DM.^2,3,4,9,10^ Thus, greater risks for DM amongst PLWHIV are consistently and significantly associated with HIV disease progression.^4,9,10^

Even though ARVs should improve the immune status of PLWHIV, however, comorbid HIV and DM aggravated by ARVs has immunosuppressive consequences that create multiple complications for the sufferers and hasten deterioration, reduce quality healthcare and may result in death.^2,11,12^ Regardless of the HIV status, obese and overweight patients should have a balanced lifestyle that aims for the loss of 5% – 7% of their initial weight in order to improve insulin sensitivity, control hyperglycaemia and maintain overall quality of life.^13,14^

The purpose of embarking on an HIV-related study is that HIV infection amongst diabetes patients has deadly outcomes, that is, poorly controlled blood sugar levels resulting in kidney disease and neuropathy.^12^ Furthermore, South Africa has the highest global HIV and DM comorbid prevalence.^2^ A study conducted in the Eastern Cape, one of its rural provinces found a 21 times significantly higher association of being diabetic for PLWHIV on protease inhibitors (PIs) when compared with those on the fixed dose combination (FDC) of tenofovir, emtricitabine and efavirenz (*p* < 0.0001); stavudine (D4T) and zidovudine (AZT) were also reported to have higher associations with diabetes in comparison to patients on FDC (*p* < 0.05).^2^ Furthermore, that same study found a threefold higher association between a longer duration on ARVs and DM (*p* < 0.005).^2^

In the context of this study, some lifestyle determinants have affected the health of individuals through the development of type 2 DM, thus increasing complications and mortalities. The study sought to investigate the lifestyle determinants of type 2 DM in the context of antiretroviral drugs to make recommendations for mitigation measures. Hence, embarking on the study to describe the predictors and determinants of DM and identify lifestyle determinants associated with type 2 DM in PLWHIV on ARV. In addition, the 2013 South African national food-based-dietary guidelines pointed out that South African communities fall short on nutrition guidelines on fruit and vegetable servings both on national and individual levels.^15^ The author further described the variance to be related to fruit and vegetable quantities and sizes that were less than 200 g, thus exposing the population to non-communicable diseases (NCDs).^15^

The findings were much less than the ideal daily fruit and vegetable servings of 400 g, an equivalence of five servings or 80 g daily that is recommended to protect against NCDs, including cancers, cardiovascular disease and type 2 DM.^16^ Such an endeavour could enhance the care standards for patients with DM and PLWHIV^17^ and also make recommendations that could align the national policies with the World Health Organization (WHO) food guidelines.^15,16^ The results of the study will also contribute to better understanding of the multi-morbidity of HIV and type 2 DM and improve knowledge on how to maintain the quality of life amongst PLWHIV receiving ARV.

## Methods

### Design

A quantitative multicentre unmatched case-control study design was used to determine the lifestyle predictors of diabetes amongst PLWHIV on ARVs. Type 2 DM patients on treatment with comorbid HIV and on antiretroviral therapy (cases) were selected and compared with PLWHIV on antiretroviral therapy without DM (controls). This study draws on the design, population and sample of participants described by Bam et al.^2^

### Setting and sample

This study was conducted in healthcare facilities of the Eastern Cape, one of South Africa’s provinces. This is the second biggest province by surface area and the fourth most populous,^18^ as it has increased from 6.6 million in 2011 to 6.7 million in 2020.^18^ It has eight health districts, OR Tambo district being the largest was conveniently chosen for this study. The study site included 10 community health centres (CHCs) and 140 clinics that had a total of 2467 patients with diabetes amongst PLWHIV.

Two controls for every case were recruited from the HIV-positive patients who were in attendance in the same clinics as the cases. Cases were identified from the primary care information system in use between the 01st of April 2015 and the 31st of March 2016. Controls were identified through a simple random sampling process of the same information system as the cases and ensured that cases and controls represented the same health facilities and sub-districts. The study was explained to them and thereafter those who met the criteria voluntarily participated after an invitation to do so was extended to them till the desired sample size was achieved. The sample size was determined using the equation:


$$\iota =\frac{{\text{Z}}_{\alpha }^{2}*P(100-P)}{e^{2}},$$ [Eqn 1]

where *n* was the minimum sample size; *Z*_α_ for a 95% confidence interval is a constant (1.96); *p* the anticipated proportion diabetes prevalence amongst PLWHIV is 8%^4^; and *e* is the maximum error accepted by the researchers at 4%.

This sample size of 177 represents the number of cases and thus 354 controls were recruited.

To be included, potential cases had to be at least 18 years old with type 2 DM on treatment and on ARVs for no less than 12 months. Cases and controls were unmatched in this study. We excluded patients with a history of defaulting ARVs on at least three different occasions.

### Data collection

A structured, standardised and validated self-administered questionnaire survey that was adapted from the WHO Stepwise tool-2016 was translated from English to isiXhosa and administered to consenting individuals. The questionnaire enquired on participants’ socio-demographic characteristics, physical activity, dietary practices and medical history. Socio-demographic factors measured include the level of education and marital status. Levels of physical activities included the types of work, recreational exercise and sport activities. Dietary practices assessed details on the intakes of fruit and vegetables and related dietary practices. Medical history included the arterial blood pressure (BP), medication including but not limited to ARVs and their complications, weight and height. Other objective measures include the height in centimetres, weight in kilograms and upper arm BP using a digital stadiometer (seca 787) and a digital BP monitor (Omron HEM-7321-E), respectively. Notwithstanding, medical records were used for the confirmation of participants’ clinical records. The study was piloted in full on three primary care attendees in one of the communities that was eventually used for the study.

### Data analysis

Data were captured in Microsoft Excel Office 16 and analysed using Stata 16.1 (Stata Corp., Lakeway Drive USA) was used for data management and analysis. The Shapiro–Wilk test was used to explore the normality of numerical data.^19^ As a result of non-normality of the distribution they were summarised using medians, 25th percentile and 75th percentile (interquartile range). Categorical variables were described using frequency tables and percentages. The Chi-squared test was used to compare two categorical variables. However, if 20% or more of the expected frequencies were less than 5 or observed frequencies less than 1 then the Fisher’s exact test was used. The Mann–Whitney *U* test was used to compare the differences of two medians. Logistics regression analyses were used to report lifestyle determinants or predictors of type 2 DM. Purposeful selection of variables was undertaken to determine the best fitting model for inclusion in the multivariable model.^2,20^ An odds ratio (OR) is the relative measure of association used in this study and the 95% confidence intervals (95% CI) is used to report on the precision of estimates. The multivariable logistics regression analyses were adjusted for age and BMI as known confounders. For statistical significance, the *p*-value was set at less than or equal to 5% (*p* ≤ 0.05).

### Ethical considerations

Formal ethical approval was sought with the Research Ethics Committee of Walter Sisulu University (075/2016). Gate – keeper permission to conduct the research was obtained from the Eastern Cape Department of Health and OR Tambo health district management and sub-districts. Research participants were informed about the purpose and objectives of the study prior to consenting to the study. The consent was sought by means of a letter and the participants were informed of their right to withdraw from the study at any time without penalty. The ethical principles that were applied in this study were respect of participants by obtaining permission, freedom to choose participation, anonymity and confidentiality, beneficence, privacy and dignity of the research participants.

## Results

A total of 177 (33.3%) of cases and 354 (66.7%) of controls were recruited into the study. Table 1 summarises demographic characteristics of participants and shows that there was no statistical difference between the sex of cases and controls (*p* = 0.927); cases and controls had statistical differences when stratified by the level of education (*p* = 0.030), participation in moderate sport (*p* = 0.007 and undertaking vigorous work (*p* = 0.019). There were statistical differences on the number of years on ARVs between cases and controls (*p* < 0.0001).

**TABLE 1:** Demographic characteristics.

Demographic characteristic	Cases diabetic (*N* = 177)			Controls non-diabetic (*N* = 354)			*P*
	*n*	%	Median (p25–p75)	*n*	%	Median (p25–p75)	
**Sex**							
Female	154	87.0	-	309	87.3	-	0.927
Male	23	13.0	-	45	12.7	-	
Age (years)	37	-	31–46	38	-	31–46	0.882
**Age (years)**							
< 40	100	56.5	-	196	55.4	-	0.943
40–49	48	27.1	-	101	28.5	-	
≥ 50	29	16.4	-	57	16.1	-	
**Highest education attained**							
Primary	109	61.6	-	204	57.6	-	0.030
Secondary	37	20.9	-	108	30.5	-	
Tertiary	31	17.5	-	42	11.9	-	
**Marital status**							
Never married	93	52.5	-	220	62.2		0.135
Married	40	22.6	-	76	21.5		
Widowed	17	9.6	-	17	4.8		
Separated	11	6.2	-	18	5.1		
Divorced	4	2.3	-	9	2.5		
Cohabiting	12	6.8	-	14	4.0		
**Years on ARVs**							
≤ 1	7	4.0	-	30	8.5	-	<0.0001
2–5	75	42.4	-	206	58.2	-	
6–10	87	49.2	-	110	31.1	-	
> 10	8	4.5	-	8	2.3	-	
**Participation in moderate sport**							
Yes	158	89.3	-	283	79.9	-	0.007
No	19	10.7	-	71	20.1	-	
**Vigorous sport**							
Yes	63	35.6	-	122	34.5	-	0.797
No	114	64.4	-	232	65.5	-	
**Vigorous work**							
Yes	70	39.6	-	178	50.3	-	0.019
No	107	60.5	-	176	49.7	-	
BMI kg/m^2^	25	-	23–29	25	-	23–29	0.290

ARV, antiretroviral treatment; BMI, body mass index; p25 = 25th percentile; p75 = 75th percentile.

Table 2 shows the vegetables and fruit eating habits of participants and shows a statistical difference in the daily fruit servings of cases and controls (*p* = 0.021). On average, 85.9% (152/177) of cases and 89.8% (318/354) of controls consumed one or two fruit daily. Only 6.2% (11/177) of cases and 7.6% (27/354) of controls consumed five fruit and vegetable servings daily.

**TABLE 2:** Vegetables and fruit eating habits.

Dietary habits	Cases diabetic (*N* = 177)			Controls non-diabetic (*N* = 354)			*p*
	*n*	%	Median (p25–p75)	*n*	%	Median (p25–p75)	
**Daily vegetable servings**							
0	2	1.1	-	5	1.4	-	0.346
1	149	84.2	-	305	86.2	-	-
2	19	10.7	-	22	6.2	-	-
3	5	2.8	-	14	4.0	-	-
4	2	1.1	-	8	2.3	-	-
Daily vegetable servings	1	-	1–1	1	-	1–1	0.532
**Daily fruit servings**							
0	0	0.0	-	1	0.3	-	0.077*
1	114	64.4	-	249	70.3	-	-
2	38	21.5	-	69	19.5	-	-
3	17	9.6	-	21	5.9	-	-
4	5	2.8	-	14	4.0	-	-
5	3	1.7	-	0	0.0	-	-
Daily fruit servings	1.1	-	0.7–2	1	-	0.6–1.7	0.091
**Daily fruit and vegetable servings**							
1	65	36.7	-	149	42.1	-	0.021
2	46	26.0	-	108	30.5	-	-
3	33	18.6	-	52	14.7	-	-
4	22	12.4	-	18	5.1	-	-
5–8	11	6.2	-	27	7.6	-	-
Daily fruit and vegetable servings	2.0	-	1–3	2.0	-	1–3	0.065

p25, 25th percentile; p75, 75th percentile.

* , Fisher’s exact test was used.

Table 3 shows that whilst 41.8% (74/177) of cases had previously been given BP advice, only 26.0% (92/354) of controls had been given BP advice (*p* < 0.0001). Other statiscally significant associations were the advice given on salt reduction (*p* = 0.021); the use of conventional antihypertensives (*p* < 0.0001) and being informed of a high BP (*p* < 0.0001).

**TABLE 3:** Blood pressure practices.

Blood pressure	Cases diabetic (*N* = 177)		Controls non-diabetic (*N* = 354)		*p*
	*n*	%	*n*	%	
**Blood pressure advice**					
Yes	5	2.8	7	2.0	0.546*
No	172	97.2	347	98.0	
**Informed of high BP**					
Yes	74	41.8	92	26.0	< 0.0001
No	103	58.2	262	74.0	
**Conventional antihypertensives used**					
Yes	69	39.0	73	20.6	< 0.0001
No	108	61.0	281	79.4	
**Advised to reduce salt intake**					
Yes	26	14.7	29	8.2	0.021
No	151	85.3	325	91.8	
**BP treated by traditional healer**					
Yes	5	2.8	8	2.3	0.768*
No	172	97.2	346	97.7	

BP, blood pressure.

* , Fisher’s exact test used.

Table 4 shows the univariable and adjusted multivariable predictors of DM. Univariable analysis showed statistically significant associations of primary education (*p* = 0.048), tertiary education (*p* = 0.012); participation in moderate sport (*p* = 0.008); undertaking vigorous work (*p* = 0.020); a report of having an average of four fruit and vegetable servings per day (*p* = 0.003); previously being informed of having a high BP and being on conventional antihypertensives (*p* < 0.0001) and being previously given advice on the need for dietary salt reduction (*p* = 0.021). In addition, there was a 3.4 and 4.3-fold higher association with diabetes for those who were on ARVs for 6–10 years (*p* = 0.006) and more than 10 years, respectively, (*p* = 0.026), in comparison to participants who had only been on ARVs for 12 months or less and these were statistically significant.

**TABLE 4:** Univariable and adjusted multivariable predictors of diabetes mellitus.

Predictor	*N*	*n*	OR	95%CI	*p*	aOR	95% CI	*p*
**Age (years)**	354	177	−0.001†	−0.9–0.02	0.913	0.01†	−0.01–0.03	0.246
**Education attained**								
Secondary	145	37	ref	-	-	ref	-	-
Primary	313	109	1.6	1.0–2.4	0.048	1.4	0.9–2.3	0.177
Tertiary	73	31	2.2	1.2–3.9	0.012	2.0	1.1–3.9	0.030
**Years on ARVs**								
≤ 1	37	7	ref	-	-	ref	-	-
2–5	281	75	1.6	0.7–3.7	0.313	1.4	0.6–3.5	0.461
6–10	197	87	3.4	1.4–8.1	0.006	3.2	1.3–8.1	0.012
> 10	16	8	4.3	1.2–15.4	0.026	3.7	0.9–14.9	0.063
**Moderate sport**								
No	90	19	ref	-	-	ref	-	-
Yes	441	158	2.1	1.2–3.6	0.008	1.9	1.1–3.4	0.030
**Vigorous work**								
No	283	107	ref	-	-	ref	-	-
Yes	248	70	0.6	0.4–0.9	0.020	0.6	0.4–0.8	0.004
**Daily fruit and vegetable servings**								
1	214	65	ref	-	-	ref	-	-
2	154	46	1.0	0.6–1.5	0.917	1.0	0.6–1.6	0.996
3	85	33	1.5	0.9–2.5	0.161	1.5	0.8–2.6	0.173
4	40	22	2.8	1.4–5.6	0.003	3.2	1.5–6.8	0.002
5–8	38	11	0.9	0.4–2.0	0.860	1.1	0.5–2.6	0.748
**Informed of high BP**								
No	365	103	ref	-	-	ref	-	-
Yes	166	74	2.0	1.4–3.0	< 0.0001	0.7	0.3–2.2	0.579
**Conventional antihypertensives**								
No	389	108	ref	-	-	ref	-	-
Yes	142	69	2.5	1.7–3.7	< 0.0001	3.1	1.0–9.5	0.049
**Advice on salt reduction**								
No		151/476	ref	-	-	ref	-	-
Yes		26/55	1.9	1.1–3.4	0.022	1.3	0.6–2.6	0509
**BMI**								
≤ 25		93/297	ref	-	-	ref	-	-
26–29		42/111	1.3	0.8–2.1	0.213	1.3	0.8–2.2	0.256
30–60		42/123	1.1	0.7–1.8	0.572	0.9	0.6–1.5	0.776

OR, odds ratio; aOR, adjusted odds ratio; BMI, body mass index; BP, blood pressure; ARVs, antiretrovirals; ref, reference.

† , Values represented are coefficients.

Adjusted multivariable analysis showed that those reporting to have a tertiary education were twice more likely to be diabetic when compared with those with secondary education and this was statistically significant (*p* = 0.030). Other statistically significant associations include those participating in moderate sport, as they were 90% probable to be diabetic (*p* = 0.030); those reporting to be on ARVs for 6 to 10 years were 3.2 times more likely to be diabetic than those who had only been on ARVs for a year or less (*p* = 0.012) and those who reported having an average of four fruit and vegetable servings daily were 3.2 times more likely to be diabetic in comparison to those who reported having one daily fruit and vegetable servings (*p* = 0.002). Furthermore, those who reported undertaking vigorous work were 40% less likely to be diabetic compared with those who did not and this was statistically significant (*p* = 0.004).

## Discussion

South Africa has shown great success in controlling the spread of HIV.^21^ The South African District Health Barometer (DHB) 2019/2020 reported a high number of adults whose viral loads were suppressed successfully per year.^21^ To the contrary, the country has seen prevalent type 2 DM amongst PLWHIV.^2,21^ This study, therefore, aimed to evaluate the lifestyle determinants of type 2 DM in the context of HIV or AIDS in the Eastern Cape province, one of South Africa’s rural provinces. The findings of the study will not only add value to research practices but are also significant for health promoters, clinicians and regulators to refine knowledge, implement informed practices and enhance policies that will elevate the standard of care for PLWHIV.

The global report on type 2 DM describes the lifestyle determinants of DM as the lifestyle factors that contribute to the onset of type 2 DM, which include unhealthy diets, physical inactivity, smoking, alcohol, ethnicity and age.^22^ The report further explains that these factors are important in the prevention of type 2 DM. The results of this study were able to reveal the lifestyle determinants of type 2 DM amongst the PLWHIV on ARVs within the rural CHCs and clinics in South Africa. The multivariate analysis illustrated significant statistical differences between the cases and controls in the type 2 DM predictors such as level of education, physical activities, dietary habits and prolonged years on ARVs. The results show the need for routine screening and assessment of the lifestyle determinants of type 2 DM amongst PLWHIV on ARVs.^4^

Furthermore, the participants who took part in vigorous work had 40% decreased probability of type 2 DM compared with those who did not engage in vigorous activities (*p* = 0.004). In this study the respondents reported participation in vigorous work three to four days per week for approximately an hour and included lifting heavy objects or digging or construction work, which increased breathing and heart rate for at least 10 min continuously. The results are consistent with those of the 2015 American Heart Association recommendations of moderate to vigorous work activity of three to four days per week to protect against disease like type 2 DM.^23^

Significant contradictions were observed on moderate sport. For example, the participants who reported participation in moderate sport such as soccer had 90% likelihood of developing type 2 DM compared with those who did not (*p* = 0.030). In addition, other contradictions noticed were on dietary habits. Participants who reported eating four fruit and vegetable servings daily had greater probability of type 2 DM than those who only ate one serving per day (*p* = 0.002). The majority of participants in this study did not meet the WHO minimal guidelines of eating about two or more servings of fruit and vegetables servings per day, which is equivalent to a maximum of 400 g or five fruit and vegetable servings per day.^16^ This is contrary to a study by Frank et al. in low- and middle-income countries, which assessed participants from the age of 15 and above and found that older individuals were more likely to adhere to the recommended guidelines compared with younger individuals and females were more likely to comply with the guidelines than males.^16,24^ However, this study could not find any statistical difference between males and females and they still became more diabetic than their counterparts who had only one fruit and vegetable serving per day. This is unfortunately the limitation of self-reported data.

The WHO guidelines on fruit and vegetable serving further stipulate that adherence to the recommended daily servings could possibly prevent the occurrence of NCDs including type 2 DM.^25^ Furthermore, in order to achieve successful self-monitoring amongst diabetic patients, motivation is critical for effective self-monitoring and control of type two DM.^26^ These authors add that absence or lack of motivation in the form of supportive close friends or family members has negative consequences that may tamper with effective self-monitoring resulting in failure to adhere to DM control measures as observed in the study.^26^ It was not in the scope of this study to measure and monitor the fruit and vegetable quantities for the participants, but sought to evaluate the lifestyle determinants of type 2 DM. Future research needs to focus on the physical and dietary determinants of type 2 DM and particularly assess the fruit and vegetable measurements for individualised counselling on balanced nutrition for patients on ARVs.^27^

Antiretroviral drugs were significant lifestyle determinants of type 2 DM. The participants who were on ARVs for 6 to 10 years were 3.2 times more likely of being diabetic than those who were only on treatment for a year, and this was statistically significant (*p* = 0.012). The results are aligned with existing research amongst PLWHIV receiving ARVs.^2,4^ Notwithstanding the given findings, long-term prospective studies to establish causality would need to be carried out to confirm the causal link and mechanism of action between antiretroviral drugs and DM.^2^

This study had a few limitations. Firstly, this is a case-control study that could not ascertain causality but could only ascertain the existence of associations. Secondly, the information on exercise and nutrition was not triangulated. This could have helped establish certainty in the association between exercise and nutrition amongst PLWHIV. These could therefore be subjects of future qualitative and quantitative prospective studies.

## Conclusion

The study found that the traditional risk factors such as a lack of physical activity, high BP, and a high BMI still play a role in the development of type 2 DM in the context of PLWHIV. In the diabetic-only population, however, the traditional risk factors seem ineffective as the odds of type 2 DM remained high despite vigorous sporting activity. The odds of type 2 DM were higher amongst the participants with tertiary education than those with primary education. Furthermore, the participants who were widows, separated, in cohabitation or married had higher probabilities of type 2 DM than those who were never married. Further research is therefore imperative to establish the specific issues that predispose married people, widows and people in cohabitation relationships to type 2 DM in order to tailor their care needs accordingly. In the context of HIV and AIDS moderate to vigorous work and sporting activity with increased intensity of at least three to four days per week is recommended not only to strengthen the heart muscle but also control type 2 DM.
